# Recombinant Human Follicle-Stimulating Hormone Alfa Dose Adjustment in US Clinical Practice: An Observational, Retrospective Analysis of a Real-World Electronic Medical Records Database

**DOI:** 10.3389/fendo.2021.742089

**Published:** 2021-12-09

**Authors:** Mary C. Mahony, Brooke Hayward, Gilbert L. Mottla, Kevin S. Richter, Stephanie Beall, G. David Ball, Thomas D’Hooghe

**Affiliations:** ^1^ North America Medical Affairs, EMD Serono, Inc. (an affiliate of Merck KGaA), Rockland, MA, United States; ^2^ Obstetrics and Gynecology, Reproductive Endocrinology and Infertility, Shady Grove Fertility Reproductive Science Center, Rockville, MD, United States; ^3^ Fertility Science Consulting, Silver Spring, MD, United States; ^4^ Seattle Reproductive Medicine, Seattle, WA, United States; ^5^ Merck Healthcare KGaA, Darmstadt, Germany; ^6^ Reproductive Medicine, Department of Development and Regeneration, Organ Systems, Group Biomedical Sciences, KU Leuven (University of Leuven), Leuven, Belgium; ^7^ Department of Obstetrics and Gynecology, Yale University, New Haven, CT, United States

**Keywords:** dose adjustments, ovarian stimulation, assisted reproductive technologies, r-hFSH- alfa, infertility

## Abstract

**Purpose:**

To determine the pattern of dose adjustment of recombinant human follicle-stimulating hormone alfa (r-hFSH-alfa) during ovarian stimulation (OS) for assisted reproductive technology (ART) in a real-world setting.

**Methods:**

This was an observational, retrospective analysis of data from an electronic de-identified medical records database including 39 clinics in the USA. Women undergoing OS for ART (initiated 2009–2016) with r-hFSH-alfa (Gonal-f^®^ or Gonal-f RFF Redi-ject^®^) were included. Assessed outcomes were patients’ baseline characteristics and dosing characteristics/cycle.

**Results:**

Of 33,962 ART cycles, 13,823 (40.7%) underwent dose adjustments: 23.4% with ≥1 dose increase, 25.4% with ≥1 dose decrease, and 8.1% with ≥1 increase and ≥1 decrease. Patients who received dose adjustments were younger (mean [SD] age 34.8 [4.58] years versus 35.9 [4.60] years, p<0.0001) and had lower BMI (25.1 [5.45] kg/m^2^ versus 25.5 [5.45] kg/m^2^, p<0.0001) than those who received a constant dose. The proportion of patients with non-normal ovarian reserve was 38.4% for those receiving dose adjustment versus 51.9% for those with a constant dose. The mean (SD) number of dose changes/cycle was 1.61 (0.92) for cycles with any dose adjustment, 1.72 (1.03) for cycles with ≥1 dose increase, 2.77 (1.00) for cycles with ≥1 dose increase and ≥1 decrease (n=2,755), and 1.88 (1.03) for cycles with ≥1 dose decrease.

**Conclusions:**

Dose adjustment during OS is common in clinical practice in the USA and occurred more often in younger versus older patients, those with a high versus non-normal ovarian reserve or those with ovulation disorders/polycystic ovary syndrome versus other primary diagnoses of infertility.

## Introduction

In the USA, recombinant human follicle-stimulating hormone alfa (r-hFSH-alfa; follitropin alfa; Gonal-f^®^ or RFF Redi-ject^®^, EMD Serono, Inc., Rockland, MA, USA, an affiliate of Merck KGaA) is used for ovarian stimulation (OS) to assist the development of multiple follicles as part of an assisted reproductive technology (ART) treatment or ovulation induction in oligoanovulatory women (1). In the case of OS, individualization of the gonadotropin starting dose is required to optimize safety and efficacy outcomes, and subsequent dose adjustments during the treatment cycle may also be needed (2–7).

The selection of the starting gonadotropin dose for OS can be dependent on specific diagnoses of subfertility and is usually based on ovarian reserve biomarkers and other baseline characteristics of the patient (2, 8–13). To this end, nomograms have been developed to guide individualization of the gonadotropin starting dose according to patient’s age and baseline serum FSH, anti-Müllerian hormone (AMH), or antral follicle count (AFC) (11, 14). Some nomograms also include additional factors, such as body mass index (BMI) or ultrasound markers (15, 16). For women with predicted hyper-response to OS, individualization of r-hFSH starting dose is advised to limit the risk of ovarian hyperstimulation syndrome (OHSS) and consequent cycle cancellation (7, 14, 15, 17, 18). For women with a predicted suboptimal response or poor response, selecting the optimal r-hFSH starting dose may reduce the risk of cycle cancellation due to inadequate response to OS (19, 20).

Dose adjustments during the cycle are usually made on the basis of individual patient’s response to stimulation, as assessed by ultrasound monitoring of follicular development, with or without assessment of hormone levels (3, 4, 13). According to the 2019 guideline of the European Society of Human Reproduction and Embryology (ESHRE), the addition of hormonal panel monitoring to the conventional ultrasound assessments during OS is probably not recommended, as it does not appear to improve fertility outcomes (13, 21). However, there was cause for concern over the validity of this conditional recommendation for OHSS risk management: only 781 women from six head-to-head comparative studies were included for analysis with ~4% OHSS rate (21). Indeed, it is acknowledged that conditional recommendations are based on weak evidence and treatment decisions may differ for each individual patient. Monitoring of hormonal profile during OS in addition to ultrasound helps to guide intra-cycle dose adjustments, which may in turn reduce the occurrence of OHSS in patients with unexpected hyper-response and potentially reduce the risk of cycle cancellations in patients with unexpected low ovarian response (21–25).

According to the prescribing information, intra-cycle dose adjustments are advised for the majority of FSH preparations, including follitropin alfa and follitropin beta (1, 26). As a result, in many randomized controlled trials comparing different gonadotropin preparations with respect to clinical outcomes, such as number of oocytes, and pregnancy and live birth rates, dose adjustments were allowed after ≥5 days of OS (27–33), and a similar dose adjustment policy has also been applied in other studies (34, 35). On the other hand, available literature suggests that maintaining a constant r-hFSH dose for the full duration of OS should be sufficient and that intra-cycle dose adjustments are generally not recommended (13, 17, 19, 36). However, such recommendations are based on the assumptions that there is no difference in efficacy outcomes in patients receiving dose adjustments versus those receiving a constant dose, and they do not take into the account the potential benefits in terms of cycle cancellation and OHSS, as well as costs or patient preferences. The benefits of intra-cycle dose adjustment are hard to assess in clinical trials as, in theory, the selection of the appropriate starting dose should lead to fewer patients with unexpected ovarian response needing adjustment during stimulation (16, 37–40). Despite optimization of the starting dose, dose adjustments are common in clinical practice and have been reported in up to 45% of ART cycles included in a recent systematic review of clinical studies (41). However, to the best of our knowledge, there are no studies assessing real-world data regarding the prevalence of dose adjustment during gonadotropin stimulation or the characteristics of patients receiving dose adjustment.

Owing to this, the aim of this analysis was to determine the pattern of dose adjustment (increase/decrease) of r-hFSH-alfa (follitropin alfa) during OS in 33,962 ART cycles in the USA and to determine the pattern of dose adjustment in different patient groups using information obtained from an electronic medical records (EMR) database.

## Materials and Methods

### Data Source

The data were obtained from a secondary, non-randomized, observational, retrospective analysis of a large, real-world, EMR database (IntegraMed America, Inc.). The clinical data set consisted of summarized de-identified clinical and laboratory data derived from a standardized EMR system for female patients who underwent fertility treatment in the USA between 1 July 2009 and 31 December 2016. The data were collected from a network of 15 practices all using the same EMR system: the practices comprised 39 clinics with 153 locations across the USA and included patients from all 50 states. Ethics Committee/Institutional Review Board approval was not required as this analysis was based on data from a de-identified EMR database.

### Patient Profiles

Data were collected from patients receiving either a r-hFSH-alfa preparation (either Gonal-f^®^ or Gonal-f^®^ RFF Redi-ject) with or without added luteinizing hormone (LH)-like-activity (i.e., Menopur [Ferring Pharmaceuticals, Saint-Prex, Switzerland] or micro-dose human chorionic gonadotropin [no product was specified in the database and this was recorded as diluted hCG]) during OS in ART cycles. Dose adjustment was defined as a change in r-hFSH-alfa dose after the start of OS and was assessed during the stimulation course, regardless of whether or how other gonadotropins were used in combination with r-hFSH-alfa during the same cycle. If r-hFSH-alfa treatment was combined with other gonadotropins, dose adjustments in gonadotropins other than r-hFSH-alfa were not taken into account. Patients’ baseline characteristics were analyzed and dosing characteristics per cycle were summarized by daily dosing pattern during OS. The dosing patterns were summarized according to cycles with a constant dose, cycles with at least one dose adjustment, cycles with at least one dose increase, regardless of any decrease, cycles with at least one dose decrease, regardless of any increase, and the subset of cycles with at least one dose increase and one dose decrease.

### Patient-Level and Cycle-Level Data

The first cycle per patient was considered as the baseline; therefore, only patient characteristics and dosing patterns for the first cycle were considered for the patient-level analysis. The patient baseline characteristics summarized by dosing pattern on their first cycle were: age, BMI, and ovarian reserve (any evidence of non-normal ovarian reserve [AFC <12 or Day 3 FSH >10 mIU/mL or AMH <1.0 ng/mL] versus normal). An AFC <12 was considered non-normal as, previously, women with a normal response to an IVF cycle had a mean AFC equal to 12 (42). The data evaluated by dosing pattern per cycle were: starting r-hFSH-alfa dose, ending r-hFSH-alfa dose, mean r-hFSH-alfa dose, minimum r-hFSH-alfa dose, and total r-hFSH-alfa dose (in IUs); the number of dose changes and minimum dose change (increment/decrement in IUs); and the length of cycle (in days), which included cycles that were cancelled.

Exploratory analyses were performed according to baseline AFC category (non-normal <12, normal ≥12), Day 3 FSH levels (normal ≤10, low >10), baseline AMH (very low [<0.5 ng/mL], low [0.5–<1.0 ng/mL], low/normal [1.0–<1.5 ng/mL], normal [1.5–4.0 ng/mL], high [>4.0 ng/mL]), and primary diagnosis at baseline.

It was initially planned to categorize patients by AMH level at baseline but, due to the observational nature of the study, data for baseline AMH were missing for some patients. Therefore, a composite measure was developed by the authors, combining available information on the ovarian reserve markers AFC, Day 3 FSH, and AMH, to determine whether there was any evidence for non-normal ovarian reserve (patients classified as “non-normal”) or there was no evidence for non-normal ovarian reserve (patients considered to have “normal” or “high” ovarian reserve, with the latter including potential hyper-responders). If data were missing for all three ovarian reserve markers, patients were classified as “ovarian reserve unknown”.

An exploratory analysis was conducted based on the ovarian reserve shown from the first cycle per patient in which patients were categorized according to their POSEIDON group: 1) patients aged <35 years with sufficient prestimulation ovarian reserve parameters (AFC ≥5, AMH ≥1.2 ng/mL) and not expected to have poor or suboptimal response, 2) patients aged ≥35 years with sufficient prestimulation ovarian reserve parameters (AFC ≥5, AMH ≥1.2 ng/mL) and not expected to have poor or suboptimal response, 3) patients aged <35 years with poor ovarian reserve prestimulation parameters (AFC <5, AMH <1.2 ng/mL), 4) patients aged ≥35 years with poor ovarian reserve prestimulation parameters (AFC <5, AMH <1.2 ng/mL) (43). The analysis used the composite measure rather than criteria based on a prior cycle and so assignment to POSEIDON Group 1 or 2 was only estimated.

### Statistical Analyses

Continuous variables were summarized using descriptive statistics (number of non-missing values, number of missing values, mean, standard deviation [SD], median, 25th Percentile – 75th Percentile [Q1–Q3], and minimum and maximum). Categorical variables were summarized by number of non-missing values, number of missing values, and percentages.

Overall P-values were calculated from one-way multivariable analysis of variance, including all baseline characteristics and starting r-hFSH-alfa dose for patient-level analyses, and including age, BMI, r-hFSH-alfa dosing, and length of cycle in days for cycle-level analyses. All comparisons were versus constant dose. Follow-up univariate analysis was performed for all sources of variation when multivariate statistically significant differences were found (at the alpha=0.05 level). Due to the large sample size and to account for the multiple comparisons, all other P-values were considered significant at the two-sided alpha=0.01 level. Analyses were completed using SAS^®^ software (version 9.4, Cary, North Carolina, USA).

## Results

### Overall Dose Adjustments

**Figure 1:**
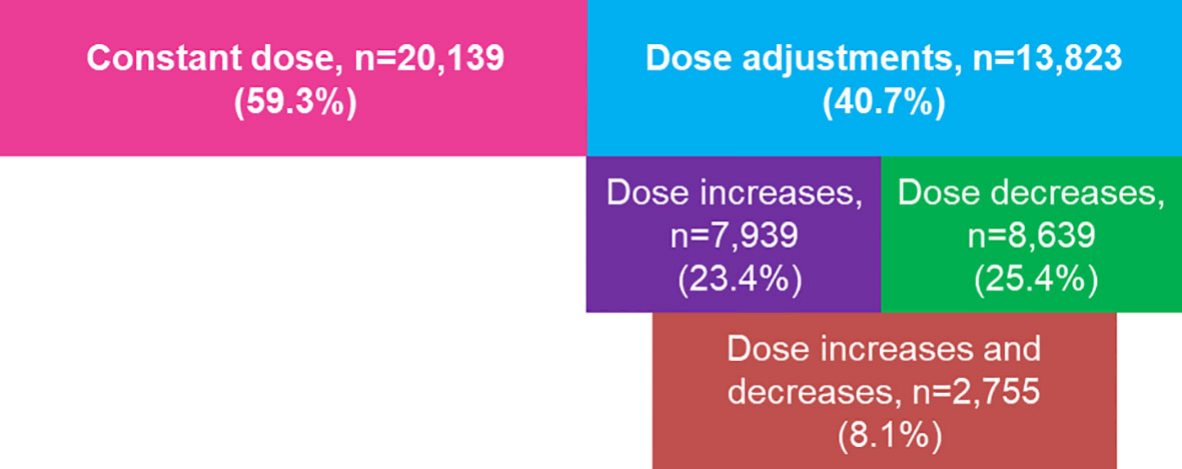
Summary of dose adjustments among cycles. Denominator for calculations is number of cycles (n=33,962). The number of cycles with: a constant dose; ≥1 dose adjustment; ≥1 dose increase, regardless of any decrease; ≥1 dose decrease, regardless of any dose increase; and ≥1 dose decrease and ≥1 increase.

Dose adjustments during OS in ART were recorded for 13,823 of 33,962 (40.7%) cycles (**Figure 1**): 23.4% had at least one dose increase, 25.4% had at least one dose decrease, and 8.1% had adjustments in both directions (at least one increase and at least one decrease).

### Patient Characteristics

The patient baseline characteristics were significantly different between patients receiving dose adjustment during OS and those receiving a constant dose (p<0.0001) (**Table 1**). Patients receiving dose adjustments were younger (mean [SD] age 34.8 [4.58] years versus 35.9 [4.60] years, p<0.0001), had a lower BMI (25.1 [5.45] kg/m^2^ versus 25.5 [5.45] kg/m^2^, p<0.0001), and a lower proportion had non-normal ovarian reserve compared with those who had a constant dose (38.4% versus 51.9%). More specifically, patients with at least one dose decrease were also younger (34.4 [4.56] years, p<0.0001), had a lower BMI (24.8 [5.24] kg/m^2^), and fewer of them had non-normal ovarian reserve (29.9%) compared with those who had a constant dose. Similarly, patients receiving at least one dose increase were younger (35.1 [4.56] years, p<0.0001) and fewer of them had a non-normal ovarian reserve (45.2%), but had no difference for BMI (25.5 [5.66] kg/m^2^, p=0.696) compared with patients who had a constant dose.

**Table 1:** Patient baseline characteristics by dosing pattern.

	All patients(N=23,582)	Constant dose(N=13,387)	Dose changes(N=10,195)	Dose increase^a^(N=5,915)	Dose decrease^b^(N=6,434)	Dose increases and decreases^c^(N=2,154)
Age (years)	35.5 (4.62)	35.9 (4.60)	34.8 (4.58)	35.1 (4.56)	34.4 (4.56)	34.3 (4.50)
P versus constant dose			<0.0001	<0.0001	<0.00al01	<0.0001
BMI (kg/m^2^)	25.3 (5.45)^d^	25.5 (5.45)^d^	25.1 (5.45)	25.5 (5.66)	24.8 (5.24)	25.2 (5.48)
P versus constant dose			<0.0001	0.6955	<0.0001	0.0207
Ovarian reserve, n (%)						
Non-normal^e^	10,854 (46.0)	6,942 (51.9)	3,912 (38.4)	2,673 (45.2)	1,923 (29.9)	684 (31.8)
Normal^f^	12,130 (51.4)	6,057 (45.2)	6,073 (59.6)	3,111 (52.6)	4,388 (68.2)	1,426 (66.2)
Unknown^g^	598 (2.5)	389 (2.9)	209 (2.1)	131 (2.2)	122 (1.9)	44 (2.0)
P versus constant dose			<0.0001	<0.0001	<0.0001	<0.0001
Race, n (%)						
White	13,561 (57.5)	7,483 (55.9)	6,078 (59.6)	3,513 (59.4)	3,856 (59.9)	1,291 (59.9)
Asian	3,792 (16.1)	2187 (16.3)	1,605 (15.7)	895 (15.1)	1,051 (16.3)	341 (15.8)
Black or African American	2,052 (8.7)	1,266 (9.5)	786 (7.7)	481 (8.1)	485 (7.5)	180 (8.4)
Hispanic or Latino	1,934 (8.2)	1,218 (9.1)	716 (7.0)	383 (6.5)	464 (7.2)	131 (6.1)
Native Hawaiian or Other Pacific Islander	124 (0.5)	65 (0.5)	59 (0.6)	32 (0.5)	38 (0.6)	11 (0.5)
American Indian or Alaska Native	79 (0.3)	49 (0.4)	30 (0.3)	16 (0.3)	19 (0.3)	5 (0.2)
Missing	2,440 (10.3)	1,358 (10.1)	1,082 (10.6)	678 (11.5)	619 (9.6)	215 (10.0)
Primary infertility diagnosis, n (%)						
Male infertility	5,020 (21.3)	2,832 (21.2)	2,188 (21.5)	1,264 (21.4)	1,405 (21.8)	481 (22.3)
Diminished ovarian reserve	4,156 (17.6)	2,651 (19.8)	1,505 (14.8)	950 (16.1)	789 (12.3)	234 (10.9)
Unexplained	3,904 (16.6)	2,161 (16.1)	1,743 (17.1)	1,020 (17.2)	1,115 (17.3)	392 (18.2)
Ovulation disorders/Polycystic ovaries	2,631 (11.2)	1,207 (9.0)	1,424 (14.0)	774 (13.1)	1,007 (15.7)	357 (16.6)
Endometriosis	1,353 (5.7)	822 (6.1)	531 (5.2)	313 (5.3)	331 (5.1)	113 (5.2)
Hydrosalpinx, in place	237 (1.0)	154 (1.2)	83 (0.8)	42 (0.7)	50 (0.8)	9 (0.4)
Tubal ligation, not reversed	294 (1.2)	185 (1.4)	109 (1.1)	60 (1.0)	63 (1.0)	14 (0.6)
Other tubal disease, no hydro	1,814 (7.7)	1,050 (7.8)	764 (7.5)	454 (7.7)	481 (7.5)	171 (7.9)
Uterine factor	422 (1.8)	278 (2.1)	144 (1.4)	84 (1.4)	87 (1.4)	27 (1.3)
Other/Unknown	3,752 (15.9)	2,048 (15.3)	1,704 (16.7)	954 (16.1)	1,106 (17.2)	356 (16.5)
P versus constant dose			<0.0001	<0.0001	<0.0001	<0.0001
Poseidon Group, n (%)^h^						
Group 1: <35 and not (AFC <5 or AMH <1.2 ng/mL)	7,717 (32.7)	3,811 (28.5)	3,906 (38.3)	2,077 (35.1)	2,752 (42.8)	923 (42.9)
Group 2: ≥35 and not (AFC <5 or AMH <1.2 ng/mL)	8,386 (35.6)	4,808 (35.9)	3,578 (35.1)	2,089 (35.3)	2,190 (34.0)	701 (32.5)
Group 3: <35 and (AFC <5 or AMH <1.2 ng/mL)	1,343 (5.7)	836 (6.2)	507 (5.0)	345 (5.8)	253 (3.9)	91 (4.2)
Group 4: ≥35 and (AFC <5 or AMH <1.2 ng/mL)	4,170 (17.7)	2,879 (21.5)	1,291 (12.7)	888 (15.0)	621 (9.7)	218 (10.1)
Missing AFC and AMH	1,966 (8.3)	1,053 (7.9)	913 (9.0)	516 (8.7)	618 (9.6)	221 (10.3)

N is the total number of patients in each category. Data are presented as mean (SD) unless stated otherwise. P-values are from models adjusted for differences in baseline characteristics and starting dose. P versus constant dose.

a Includes all patients with at least one dose increase (regardless of any decrease) in their first cycle.

b Includes all patients with at least one dose decrease (regardless of any increase) in their first cycle.

c Includes all patients with at least one dose increase and one dose decrease in their first cycle.

d Data missing for three patients.

e Non-normal ovarian reserve: antral follicle count <12 OR Day 3 follicle-stimulating hormone >10 mIU/mL OR anti-Müllerian hormone <1.0 ng/mL.

f All patients without evidence of non-normal ovarian reserve and not missing all three factors were considered to have normal ovarian reserve, including patients with high ovarian reserve and potential hyper-responders.

g Missing data for all three ovarian reserve markers (antral follicle count, Day 3 follicle-stimulating hormone and anti-Müllerian hormone).

h Based on first cycle per patient.

In patients with dose adjustments, 59.6% had normal ovarian reserve, whereas 45.2% of patients with a constant dose had normal ovarian reserve (**Table 1**). In patients with at least one dose increase, 52.6% had normal ovarian reserve, whereas 68.2% of patients with at least one dose decrease had normal ovarian reserve. Based on age, AFC and AMH before the start of the first cycle per patient, of the patients with a constant dose, 28.5% met the criteria for POSEIDON Group 1 (women younger than 35 years with normal ovarian reserve not expected to have a low ovarian response) and 21.5% met the criteria for POSEIDON Group 4 (women older than 35 years with reduced ovarian reserve and expected low ovarian response), whereas the proportions of patients with dose changes meeting the criteria for POSEIDON Groups 1 or 4 were 38.3% and 12.7%, respectively (**Table 1**). The proportions of patients with a constant dose or a dose change were similar between POSEIDON Groups 2 (range 35–36%) and 3 (range 5–6%) (**Table 1**). Overall, dose changes were observed more commonly in women with a normal ovarian reserve not expected to have a low ovarian response (38.3% for POSEIDON Group 1, 35% for POSEIDON Group 2) than in women with an abnormal ovarian reserve and expected low ovarian response (5% for POSEIDON Group 3, 12.7% for POSEIDON Group 4).

#### Dosing Characteristics

Pituitary down-regulation was performed with a gonadotropin-releasing hormone antagonist in 59.2% of cycles with constant dosing compared with 60.1% of cycles with dose adjustment. Compared with cycles where the dose was constant, cycles with at least one dose increase had a lower mean starting dose and minimum dose per cycle and a higher mean ending dose and total dose per cycle. LH-like products were used in >90% of cycles, with no difference in the number of cycles with dose changes or with a constant dose (**Table 2**).

**Table 2:** Dosing characteristics per cycle.

	All cycles(N=33,962)	Constant dose (N=20,139)	Dose changes (N=13,823)	Dose increase^a^ (N=7,939)	Dose decrease^b^ (N=8,639)	Dose increasesand decreases^c^ (N=2,755)
GnRH use, n (%)						
Agonist	13435 (39.6)	8008 (39.8)	5427 (39.3)	2835 (35.7)	3601 (41.7)	1009 (36.6)
Antagonist	20231 (59.6)	11921 (59.2)	8310 (60.1)	5036 (63.4)	4987 (57.7)	1713 (62.2)
None or unknown	296 (0.9)	210 (1.0)	86 (0.6)	68 (0.9)	51 (0.6)	33 (1.2)
Use of LH-like product, n (%)						
No, r-hFSH-alfa only	1,390 (4.1)	895 (4.4)	495 (3.6)	207 (2.6)	377 (4.4)	89 (3.2)
Yes^d^	32,572 (95.9)	19,244 (95.6)	13,328 (96.4)	7,732 (97.4)	8,262 (95.6)	2,666 (96.8)
hMG	29,639 (87.3)	17,480 (86.8)	12,159 (88.0)	7,226 (91.0)	7,431 (86.0)	2,498 (90.7)
micro-dose hCG	2,724 (8.0)	1,612 (8.0)	1,112 (8.0)	477 (6.0)	784 (9.1)	149 (5.4)
r-hLH	557 (1.6)	363 (1.8)	194 (1.4)	104 (1.3)	124 (1.4)	34 (1.2)
r-hFSH-alfa treatment characteristics						
Starting dose	275.87 (130.99)	292.47 (133.24)	251.69 (123.73)	237.76 (122.58)	257.98 (133.28)	231.28 (149.88)
Ending dose	255.33 (137.61)	265.72 (135.25)	240.20 (139.60)	294.86 (130.20)	183.15 (121.62)	218.80 (125.75)
Total dose	2,838.1 (1,537.6)	2,916.0 (1,567.9)	2,724.5 (1,485.1)	3135.4 (1,506.4)	2,271.7 (1,295.5)	2488.8 (1,345.3)
Minimum dose	227.62 (123.89)	260.01 (127.43)	191.24 (108.86)	213.52 (102.24)	160.26 (105.74)	158.31 (95.32)
Average dose	282.57 (133.40)	294.81 (137.77)	265.19 (124.89)	293.71 (121.67)	230.66 (117.46)	233.66 (112.78)

N is the number of cycles in each dosing pattern. Data are presented as mean (SD) IU unless stated otherwise.

GnRH, gonadotropin-releasing hormone.

a Includes all cycles with at least one dose increase (regardless of any decrease).

b Includes all cycles with at least one dose decrease (regardless of any increase).

c Includes all cycles with at least one dose increase and one dose decrease.

d Patients could have been prescribed >1 LH-like product.

The mean starting dose (257.98 [133.28] IU/day), ending dose (183.15 [121.62] IU/day), total dose 2,271.70 [1,295.50] IU), minimum dose (160.26 [105.74] IU), and average dose (230.66 [117.46] IU/day versus 294.81 [137.77] IU/day) were all lower in cycles where there was a dose decrease compared with those where the dose was constant (**Table 2**).

### Number of Dose Adjustments During Gonadotropin Stimulation

**Figure 2:**
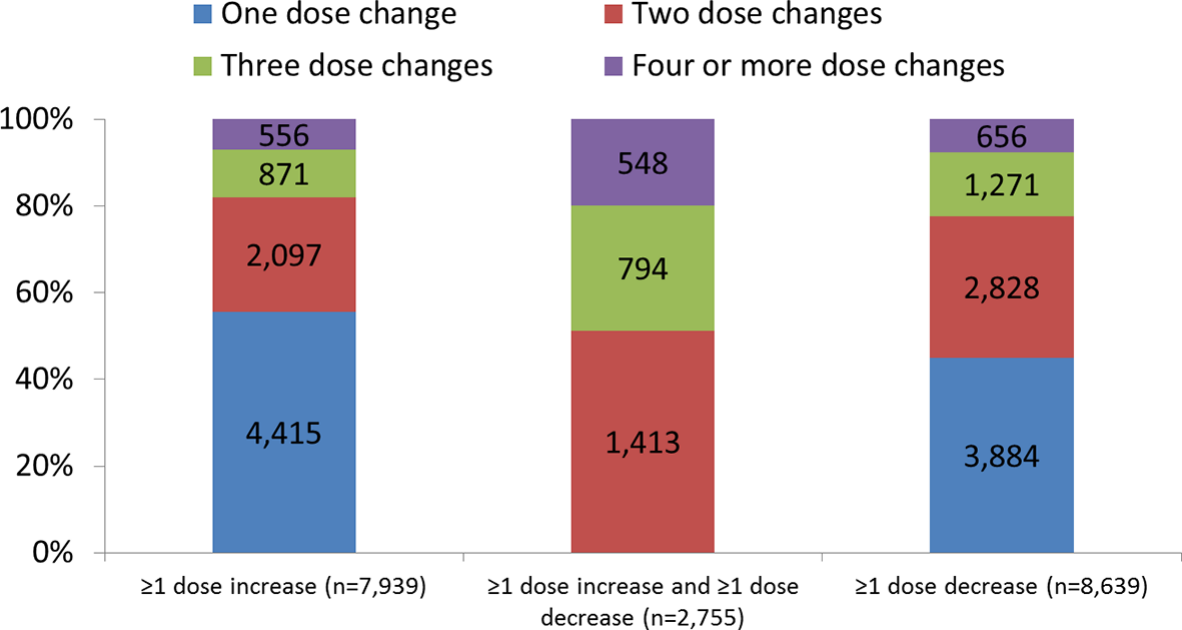
Number of dose changes per cycle. Data are presented as number and proportion in each dosing category. ≥1 dose increase includes all cycles with at least one dose increase (regardless of any decrease). ≥1 dose decrease includes all cycles with at least one dose decrease (regardless of any dose increase).

Overall, 13,823 cycles had a dose adjustment and 20,139 cycles had a constant dose (**Figure 1**). The mean (SD) number of dose changes per cycle was 1.61 (0.92) in cycles with any dose adjustment (n=13,823), 1.72 (1.03) in those with ≥1 dose increase (n=7,939), 2.77 (1.00) in those with ≥1 dose increase and ≥1 dose decrease (n=2,755), and 1.88 (1.03) in those with a dose decrease (n=8,639). Nearly two-thirds of the cycles (60%) had only one dose change: 55.6% of cycles with a single dose increase and 45.0% of cycles with a single dose decrease (**Figure 2**).

### Extent of Dose Changes

**Figure 3:**
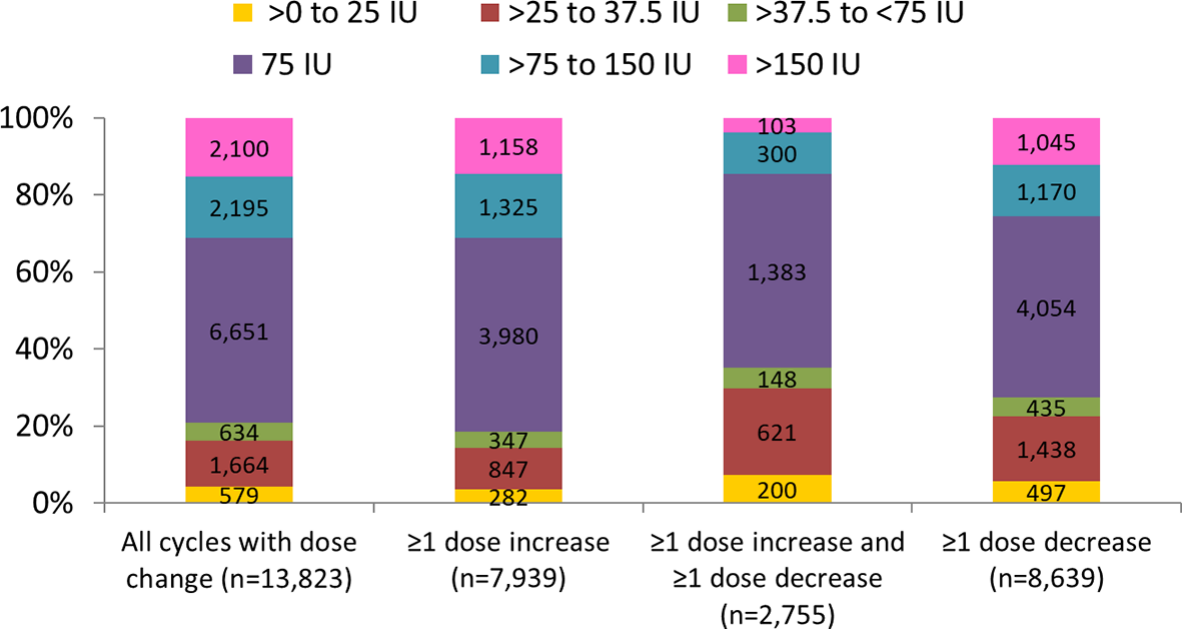
Minimum dose adjustment per cycle. Data are presented as number and proportion in each dosing category. ≥1 dose increase includes all cycles with at least one dose increase (regardless of any decrease). ≥1 dose decrease includes all cycles with at least one dose decrease (regardless of any dose increase).

The overall mean (SD) minimum dose change for all cycles with dose adjustment was 115.70 (113.25) IU: 113.82 (112.28) IU in cycles with a dose increase, 106.88 (123.78) IU in cycles with a dose decrease, and 82.61 (138.49) IU in cycles with both an increase and decrease. The largest proportion of cycles used 75 IUs as the smallest increment of dose adjustment: 48.1% of cycles had a dose change of 75 IU, 20.8% used <75 IUs as the smallest increment, and 31.1% used >75 IUs as the smallest increment (**Figure 3**). The smallest increments of dose adjustments (<75 IU) were more often used in cycles with dose decreases (27.4%) and in the subset with decreases and increases (35.2%), highlighting the practice of small dose changes (**Figure 3**).

### Length of Ovarian Stimulation

**Figure 4:**
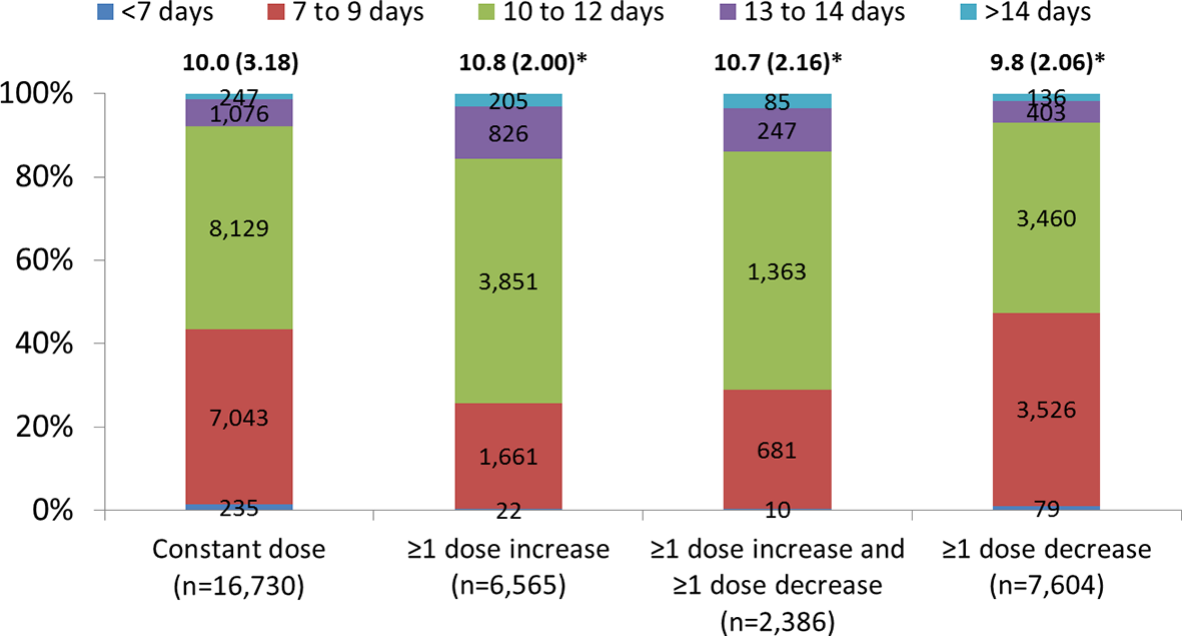
Length of ovarian stimulation (including cancelled cycles). Data are presented as mean (standard deviation) and number and proportion in each dosing category with no missing cycle length. ≥1 dose increase includes all cycles with at least one dose increase (regardless of any decrease). ≥1 dose decrease includes all cycles with at least one dose decrease (regardless of any dose increase). *p < 0.0001 compared with constant dose. P-values are from models adjusted for differences in age, body mass index, starting dose and other dosing characteristics.

Overall, the mean (SD) length of all cycles was 10.1 (2.77) days. Mean (SD) OS was longer in cycles where there was a dose increase compared with those where the dose was constant (10.8 [2.00] versus 10.0 [3.18] days, p<0.0001). The highest proportion of cycles with constant dose, dose increase, or dose increase and dose decrease had OS with a duration between 10 and 14 days; cycles with ≥1 dose decrease had a higher proportion of OS with a duration shorter than 10 days as compared to cycles with constant dose, ≥1 dose increase, or ≥1 dose increase and ≥1 dose decrease. Few cycles had OS duration of <7 days or >14 days (**Figure 4**).

### Exploratory Analyses

#### Baseline AFC

Mean (SD) baseline AFC was 15.5 (9.6) in those receiving dose adjustment compared with 13.1 (8.5) in patients receiving a constant dose (**Supplementary Table 1**). A higher proportion of patients with no dose adjustment had a non-normal AFC (<12 follicles) compared with patients with dose adjustment (42% versus 31%) (**Supplementary Table 1**).

#### Day 3 FSH Level

Mean (SD) Day 3 FSH levels were 7.4 [2.8] mIU/mL in those receiving dose decreases and 7.6 [3.6] mIU/mL in those receiving both dose increases and decreases compared with 8.2 (4.8) mIU/mL in patients receiving a constant dose (decrease: p<0.0001; increases and decreases: p=0.0018) (**Supplementary Table 1**). There was no difference between patients receiving dose increases and those receiving a constant dose (**Supplementary Table 1**).

#### Baseline AMH

Mean (SD) baseline AMH levels were 3.0 (3.8) ng/mL in those who received a dose increase, 4.0 (4.4) ng/mL in those who received a dose decrease, and 3.9 (4.6) ng/mL in those who received both increases and decreases, compared with 2.2 (2.9) ng/mL in patients who received a constant dose (**Supplementary Table 1**).

#### Primary Infertility Diagnosis at Baseline

The distribution of infertility diagnoses at baseline (before starting their first cycle) differed for women with dose adjustment compared with women with constant dosing (p<0.0001, **Table 1**). A higher proportion of women with dose adjustment compared with constant dose were undergoing OS in ART owing to ovulation disorders/polycystic ovary syndrome (PCOS; 14.0% versus 9.0%, respectively), unexplained infertility (17.1% versus 16.1%), or other/unknown factors (16.7% versus 15.3%), p<0.0001. A lower proportion of women with dose adjustment compared with constant dose were diagnosed with diminished ovarian reserve (14.8% versus 19.8%, respectively), endometriosis (5.2% versus 6.1%), hydrosalpinx (0.8% versus 1.2%), or uterine factor (1.4% versus 2.1%), p<0.0001 (**Supplementary Table 1**).

## Discussion

Results of this analysis of the real-world data from the US database show that r-hFSH-alfa dose adjustment is common during OS in ART: out of 33,962 OS cycles (23,582 patients), 40.7% had at least one dose adjustment. Among cycles with dose changes, 57.4% had at least one dose increase, 62.5% had at least one dose decrease, and 19.9% of cycles included both increases and decreases. The prevalence of r-hFSH dose adjustments during OS reported in our analysis is generally consistent with the findings of a recent systematic review of clinical studies, in which dose adjustment with unspecified direction was observed in 45% of assessed cycles (41).

Although the exact reasons for dose adjustments were not assessed in our analysis, we hypothesize that the main factors contributing to the decision to adjust the dose during the cycle were patient preferences and physician anticipation that dose modification based on ovarian response would lead to improved outcomes and/or would help avoid complications. For example, cycle cancellation due to the risk of OHSS may necessitate discontinuation of the ongoing ART treatment and/or subsequent OS cycles, which increases both the emotional and financial burden on patients (7). Evidence suggests that the discontinuation rate is higher amongst patients undergoing subsequent treatment cycles after a failed ART treatment in previous attempts (44). Dose adjustment during gonadotropin stimulation based on ovarian response to treatment measured by ultrasound assessment of follicular development and hormonal monitoring is a common strategy to limit the risk of OHSS and reduce the occurrence of cycle cancellations (18, 38, 45). In our study, patients with potential high ovarian reserve and potential hyper-responders were grouped into the “normal ovarian reserve” group since they did not show evidence of a non-normal ovarian reserve and a significant number of dose decreases was observed in this group. Additionally, cycles with dose decreases had a higher proportion of OS with a duration shorter than 10 days as compared to cycles with a constant dose, which may be due to cancelled cycles. Together, these facts may indicate that the potential hyper-responders in our analysis needed further dose decreases beyond the individualization of the starting dose to reduce the risk of OHSS.

In our analysis, patients with a dose increase had a longer duration of OS than those who had a constant dose (mean [SD] 10.8 [2.00] versus 10.0 [3.18], p<0.0001). While we did not assess follicular growth in this analysis, this observation is likely not due to the dose increase itself, but rather may be the result of the slow initial development of follicles and consequent need for a longer stimulation cycle and a dose increase during stimulation. In order to maximize the ovarian potential, reduce the time to live birth, and increase cumulative live birth rates, an optimal number of oocytes should be retrieved without putting the patient at risk for OHSS (46–49). Patients with unexpected low ovarian response demonstrate an initial slow response to FSH stimulation in terms of estradiol levels and follicle growth (25). Furthermore, unexpected low ovarian response has been linked to genetic factors that impact the ovarian response to ovarian stimulation with the gonadotropins FSH and LH. Clinically relevant polymorphisms, such as in the T allele of the FSHB promotor, can affect transcriptional activity and reduce circulating levels of the hormones, thereby impacting ovarian response (50). The FSH receptor gene has also been found to be clinically relevant, and has been identified as a promising candidate for a targeted pharmacogenetic approach to better standardize COS for women undergoing ART (51). The value of added genetic testing to ART cycles was also evaluated by Ga et al., who compared the value of LH supplementation guided by conventional methods or by polymorphisms in the LHCGRE gene. They found that by using genetics as a basis for guiding LH dose, the regimen provided optimum levels of r-hLH in patients with impaired hormone-receptor interacting activity and achieved higher pregnancy and live birth rates (52). Therefore, increasing the FSH dose during treatment in these patients may increase the number of oocytes retrieved, particularly in patients with a low follicle-to-oocyte ratio (24). This, in turn, could help reduce the risk of cycle cancellation due to the expected absence or very low yield of viable oocytes to retrieve, subsequently diminishing treatment discontinuation rates and their associated financial and emotional implications to patients (25, 49, 53). A recent expert panel has discussed the future role for genetic testing in ART, and suggested that this has a place in mainstream medicine (54).

In our study, the exploratory analysis of the patient characteristics showed that patients receiving dose adjustment (increases, decreases, and both increases and decreases) during OS had a higher baseline AFC, higher AMH levels, and a lower Day 3 FSH level compared with patients with a constant dose. This observation may be explained by the fact that patients with non-normal ovarian reserve generally start OS with a higher starting dose. Indeed, the r-hFSH-alfa starting dose in patients receiving a constant dose was around 300 IU compared with ~ 250 IU in those receiving dose adjustment during gonadotropin stimulation. The exploratory analysis of dose adjustment according to POSEIDON group was conducted using the baseline information, i.e., from before the first cycle/patient and so only uses the composite age, AFC, and AMH criteria, rather than criteria based on a response from a prior cycle, limiting the accuracy of identifying POSEIDON Groups 1 and 2. The higher proportion of dose changes among patients from POSEIDON Groups 1 (38%) and 2 (35%) – who had a normal ovarian reserve and were therefore expected to have a good response – may indicate that dose adjustments were required in this group owing to an unexpected ovarian response. Conversely, the lower proportion of dose changes among patients meeting the criteria for POSEIDON Groups 3 (5%) or 4 (12.7%) – who were expected to have a poor response – may reflect individualization of the starting dose based on age and ovarian reserve markers, resulting in a high starting dose, limiting the need for additional dose increases during ovarian stimulation. Patients who received a dose adjustment during gonadotropin stimulation were less likely to have a diagnosis of diminished ovarian reserve and were more likely to have ovulation disorders/PCOS compared with patients treated with a constant dose. Patients with ovulation disorders/PCOS may have been started on a lower dose to avoid OHSS. However, patients with diminished ovarian reserve may have had individualization of the starting dose following measurement of ovarian reserve biomarkers, and those with endometriosis, hydrosalpinx, and uterine factors may have had individualization of the starting dose as their response to ovarian stimulation may be altered by these conditions. These findings imply that dose adjustments should not be considered exclusively before or during treatment. Instead, individualization of the gonadotropin dose may require both setting the appropriate starting dose based on baseline biomarkers, as well as the option for dose adjustment during gonadotropin stimulation according to a patient response. However, more studies reporting real-world data from clinical practice are needed to assess the true value of such a flexible approach to gonadotropin dose adjustments and to distinguish the effects of dose adjustment before and/or during treatment.

The current analysis may have the limitation of being a retrospective, observational study of real-world US data that was not specifically collected for research purposes and, therefore, may not reflect clinical practices in other parts of the world. Furthermore, diagnostic and drug use information may not always be validated or complete. In addition, the scope of this analysis was restricted to the occurrence of r-hFSH-alfa dose adjustments during treatment and we did not consider reproductive outcomes, safety and cancellations, or other outcomes related to the dose adjustments during OS; therefore, further analysis should include the impact of dose adjustment on outcomes. Owing to missing data, classification of patients as non-normal ovarian reserve was made by creating a composite measure of baseline ovarian reserve biomarkers, to determine whether there was evidence or not for non-normal ovarian reserve. Measurements of AFC and AMH were conducted *via* different methods depending on the center at which they were assessed. In addition, data on the length of cycles was for all cycles started, including those that were cancelled; therefore, the cancelled cycles were included in the results for short cycles.

## Conclusions

Dose adjustment during gonadotropin stimulation, depending on ovarian response, is highly prevalent (40.7%) during OS in ART cycles in real-world practice in the USA. Dose decreases were the most common dose adjustment (more than 60% of cycles), nearly a fifth of cycles included both increases and decreases, and patients with poorer ovarian reserve started with a higher dose. Dose adjustments during gonadotropin stimulation are used for all patient types, but are more frequent in younger patients and those with higher ovarian reserve and a diagnosis of ovulation disorders/PCOS. Further analyses are warranted to determine whether dose adjustment impacts outcomes, such as the number of cycles cancelled, oocytes retrieved and embryos available for transfer/cryopreservation.

## Previous Publications

An earlier analysis of these data was presented as a poster at the 34th annual meeting (2018) of the European Society of Human Reproduction and Embryology ESHRE.

## Data Availability Statement

Any requests for data by qualified scientific and medical researchers for legitimate research purposes will be subject to Merck KGaA’s Data Sharing Policy. All requests should be submitted in writing to Merck KGaA’s data sharing portal https://www.merckgroup.com/en/research/our-approach-to-research-and-development/healthcare/clinical-trials/commitment-responsible-data-sharing.html. When Merck KGaA has a co-research, co-development, or co-marketing or co-promotion agreement, or when the product has been out-licensed, the responsibility for disclosure might be dependent on the agreement between parties. Under these circumstances, Merck KGaA will endeavour to gain agreement to share data in response to requests.

## Author Contributions

TD’H, BH, GM, KR, and MM contributed to the conception and design of the analysis. All authors contributed to the interpretation of data, data analysis and critical review of this manuscript. All authors contributed to the article and approved the submitted version.

## Funding

This study was sponsored by EMD Serono Inc., an affiliate of Merck KGaA (CrossRef Funder ID: 10.13039/100004755).

## Conflict of Interest

BH is an employee of EMD Serono, Inc., Rockland,MA, USA (an affiliate of Merck KGaA). TD’H is an employee of Merck KGaA, Darmstadt, Germany. At the time of the analysis, MM was an employee of EMD Serono, Inc., Rockland, MA, USA (an affiliate of Merck KGaA). KR is Founder and Chief Scientist of the company Fertility Science Consulting.

This study was sponsored by EMD Serono Inc., an affiliate of Merck KGaA (CrossRef Funder ID: 10.13039/100004755). The sponsor designed and approved the study, took part in data analysis, and contributed to the data interpretation and final draft of the manuscript. The corresponding author had full access to all the data in the study and had final responsibility for the decision to submit for publication.

## Publisher’s Note

All claims expressed in this article are solely those of the authors and do not necessarily represent those of their affiliated organizations, or those of the publisher, the editors and the reviewers. Any product that may be evaluated in this article, or claim that may be made by its manufacturer, is not guaranteed or endorsed by the publisher.
