# Supplementary material for: Recombinant Human Follicle-Stimulating Hormone Alfa Dose Adjustment in US Clinical Practice: An Observational, Retrospective Analysis of a Real-World Electronic Medical Records Database
Source: Front Endocrinol (Lausanne). 2021 Dec 9;12:742089. doi: 10.3389/fendo.2021.742089 (PMC8696034; doi:10.3389/fendo.2021.742089)
Supplement: Supplementary file 1 [file Table_1.docx]

**Supplementary materials**

**Supplementary table 1: Baseline AFC, Day 3 FSH, baseline AMH and initial diagnosis**

| **Baseline characteristics** | **All patients**  **(N=23,582)** | **Constant dose**  **(N=13,387)** | **Dose changes**  **(N=10,195)** | **Dose increase^a^**  **(N=5,915)** | **Dose decrease^b^**  **(N=6,434)** | **Dose increases and decreases^c^**  **(N=2,154)** |
| --- | --- | --- | --- | --- | --- | --- |
| **AFC by dosing pattern** | **(n=18,122)** | **(n=10,553)** | **(n=7,569)** | **(n=4,437)** | **(n=4,701)** | **(n=1,569)** |
| Baseline AFC | 14.1 (9.08) | 13.1 (8.52) | 15.5 (9.64) | 14.1 (9.20) | 17.3 (10.05) | 17.0 (10.35) |
| P versus constant dose |  |  | <0.0001 | <0.0001 | <0.0001 | <0.0001 |
| AFC by category, n (%) | | | | | | |
| Non-normal (<12) | 8,695 (37%) | 5,567 (42%) | 3,128 (31%) | 2,155 (36%) | 1,504 (23%) | 531 (25%) |
| Normal (≥12) | 9,427 (40%) | 4,986 (37%) | 4,441 (43%) | 2,282 (39%) | 3,197 (50%) | 1,038 (48%) |
| Missing | 5,460 (23%) | 2,835 (21%) | 2,625 (26%) | 1,478 (25%) | 1,732 (27%) | 585 (27%) |
| P versus constant dose |  |  | <0.0001 | <0.0001 | <0.0001 | <0.0001 |
| **Day 3 FSH level** | **(n=20,045)** | **(n=11,490)** | **(n=8,555)** | **(n=4,923)** | **(n=5,457)** | **(n=1,825)** |
| Day 3 FSH (mIU/mL) | 8.0 (4.37) | 8.2 (4.81) | 7.8 (3.70) | 8.1 (4.41) | 7.4 (2.82) | 7.6 (3.57) |
| P versus constant dose |  |  | <0.0001 | 0.3570 | <0.0001 | 0.0018 |
| Day 3 FSH by category, n (%) | | | | | | |
| Normal (≤10 mIU/mL) | 18,272 (78%) | 10,308 (77%) | 7,964 (78%) | 4,493 (76%) | 5,207 (81%) | 1,736 (81%) |
| High (>10 mIU/mL) | 1,773 (7%) | 1,182 (9%) | 591 (6%) | 430 (7%) | 250 (4%) | 89 (4%) |
| Missing | 3,537 (15%) | 1,898 (14%) | 1,639 (16%) | 992 (17%) | 976 (15%) | 329 (15%) |
| P versus constant dose |  |  | <0.0001 | 0.0240 | <0.0001 | <0.0001 |
| **AMH by dosing pattern** | **(n=12,312)** | **(n=6,684)** | **(n=5,628)** | **(n=3,148)** | **(n=3,600)** | **(n=1,120)** |
| Baseline AMH, ng/mL | 2.8 (3.56) | 2.2 (2.90) | 3.5 (4.09) | 3.0 (3.80) | 4.0 (4.43) | 3.9 (4.56) |
| AMH by category, n (%) | | | | | | |
| Very Low  (<0.5 ng/mL) | 2,138 (9%) | 1,487 (11%) | 651 (6%) | 470 (8%) | 286 (4%) | 105 (5%) |
| Low  (0.5–<1.0 ng/mL) | 1,922 (8%) | 1,287 (9%) | 635 (6%) | 420 (7%) | 332 (5%) | 117 (5%) |
| Low Normal  (1.0–<1.5 ng/mL) | 1,488 (6%) | 865 (7%) | 623 (6%) | 377 (6%) | 363 (6%) | 117 (5%) |
| Normal  (1.5–4.0 ng/mL) | 4,252 (18%) | 2,099 (16%) | 2,153 (21%) | 1,153 (20%) | 1,416 (22%) | 416 (19%) |
| High  (>4.0 ng/mL)) | 2,512 (11%) | 946 (7%) | 1,566 (15%) | 728 (12%) | 1,203 (19%) | 365 (17%) |
| Missing | 11,270 (48%) | 6,703 (50%) | 4,567 (46%) | 2,767 (47%) | 2,834 (44%) | 1,034 (49%) |
| P versus constant dose |  |  | <0.0001 | <0.0001 | <0.0001 | <0.0001 |
| N is the total number of patients in each dosing pattern, n is the number of patients with reported/missing data. Data are presented as mean (SD) unless stated otherwise. P-values are from models adjusted for differences in baseline characteristics and starting dose. AFC category proportions calculated from first cycle data with total for all patients in each category as the denominator. AFC, antral follicle count. AMH, anti-Müllerian hormone. FSH, follicle-stimulating hormone.  a. Includes all patients with at least one dose increase (regardless of any decrease) in their first cycle.  b. Includes all patients with at least one dose decrease (regardless of any increase) in their first cycle.  c. Includes all patients with at least one dose increase and one dose decrease in their first cycle. | | | | | | |
